# A One Base Pair Deletion in the Canine *ATP13A2* Gene Causes Exon Skipping and Late-Onset Neuronal Ceroid Lipofuscinosis in the Tibetan Terrier

**DOI:** 10.1371/journal.pgen.1002304

**Published:** 2011-10-13

**Authors:** Anne Wöhlke, Ute Philipp, Patricia Bock, Andreas Beineke, Peter Lichtner, Thomas Meitinger, Ottmar Distl

**Affiliations:** 1 Institute for Animal Breeding and Genetics, University of Veterinary Medicine Hannover, Hannover, Germany; 2 Department of Pathology, University of Veterinary Medicine Hannover, Hannover, Germany; 3 Helmholtz Zentrum München, Institute of Human Genetics, Neuherberg, Germany; 4 Technische Universität München, Institute of Human Genetics, München, Germany; Stanford University School of Medicine, United States of America

## Abstract

Neuronal ceroid lipofuscinosis (NCL) is a progressive neurodegenerative disease characterized by brain and retinal atrophy and the intracellular accumulation of autofluorescent lysosomal storage bodies resembling lipofuscin in neurons and other cells. Tibetan terriers show a late-onset lethal form of NCL manifesting first visible signs at 5–7 years of age. Genome-wide association analyses for 12 Tibetan-terrier-NCL-cases and 7 Tibetan-terrier controls using the 127K canine Affymetrix SNP chip and mixed model analysis mapped NCL to dog chromosome (CFA) 2 at 83.71–84.72 Mb. Multipoint linkage and association analyses in 376 Tibetan terriers confirmed this genomic region on CFA2. A mutation analysis for 14 positional candidate genes in two NCL-cases and one control revealed a strongly associated single nucleotide polymorphism (SNP) in the *MAPK PM20/PM21* gene and a perfectly with NCL associated single base pair deletion (c.1620delG) within exon 16 of the *ATP13A2* gene. The c.1620delG mutation in *ATP13A2* causes skipping of exon 16 presumably due to a broken exonic splicing enhancer motif. As a result of this mutation, ATP13A2 lacks 69 amino acids. All known 24 NCL cases were homozygous for this deletion and all obligate 35 NCL-carriers were heterozygous. In a sample of 144 dogs from eleven other breeds, the c.1620delG mutation could not be found. Knowledge of the causative mutation for late-onset NCL in Tibetan terrier allows genetic testing of these dogs to avoid matings of carrier animals. *ATP13A2* mutations have been described in familial Parkinson syndrome (PARK9). Tibetan terriers with these mutations provide a valuable model for a PARK9-linked disease and possibly for manganese toxicity in synucleinopathies.

## Author Summary

The neuronal ceroid lipofuscinosis (NCL) is a neurodegenerative storage diseases characterized by psychomotor retardation, blindness, and premature death. NCL has been reported in several dog breeds. NCL is characterized by progressive brain and retinal atrophy and the intracellular accumulation of autofluorescent lysosomal storage bodies resembling lipofuscin. Tibetan terriers show a late-onset and lethal NCL (age of onset 5–7 years) with an autosomal recessive inheritance. The most frequently described first symptom is blindness in twilight. In the disease progress the affected dogs often appear nervous or anxious and the lack of motor coordination becomes more severe. In the final stages of this disease, mild but also severe seizures have been observed by the owner. There are no treatment options for affected dogs. Through a genome-wide association analysis using the 127K canine Affymetrix SNP chip, we found a 1 Mb candidate genomic region and identified *ATP13A2* as the most likely candidate for NCL. A 1-base pair deletion mutation within exon 16 of the *ATP13A2* gene caused the loss of an exonic splicing enhancer and, consequently, the alternative splicing lead to skipping of exon 16. This study provides a suitable animal model for PARK9 in man to develop therapeutic approaches.

## Introduction

Neuronal ceroid lipofuscinosis (NCL) is a progressive neurodegenerative diseases characterized by brain and retinal atrophy and the intracellular accumulation of autofluorescent lysosomal storage bodies resembling lipofuscin in neurons and other cells. NCL has been reported in several domestic animal species including cattle, goat, sheep, cat and dog [1]. To this date eight forms of NCL have been classified by clinical criteria, age of onset and presence of lysosomal storage material in humans. Causative mutations in nine genes (PPT1, TPP1, CLN3, CLN5, CLN6, CLN7, CLN8, CTSD and CLCN6) have been identified [2]–[7]. All these mutations lead to an early-onset NCL in human, sheep or dogs. Tibetan terriers show a late-onset lethal form of NCL manifesting at 5–7 years [8]. The disease starts most frequently with blindness in twilight and disorientation. Affected dogs often appear nervous or anxious and the lack of motor coordination becomes more severe with disease progression. Affected dogs often have problems to jump up from the ground floor, or have problems by going upstairs. At the final stages of the disease mild to severe seizures have been observed. There are no treatment options for affected dogs. Due to the late-onset of NCL in Tibetan terriers some of the NCL susceptible dogs are bred and have progeny.

Tibetan terriers provide a valuable model for late-onset NCL as no causative mutation for late onset NCL is known in human and other species. NCL-genes known from human, sheep or other dog breeds were evaluated and excluded for late-onset NCL in Tibetan terriers in previous studies [9]–[13]. Parallel with our analysis, an *ATP13A2* frameshift mutation was identified as responsible for adult-onset neuronal ceroid lipofuscinosis in Tibetan terriers [14]. Here, we describe the same association of the canine chromosome (CFA) 2 region which harbors a splice-variant mutation in canine *ATP13A2* with late-onset NCL and analyse the mRNA of *ATP13A2* from NCL-affected Tibetan terriers to characterize the possible effect of this mutation on the resulting protein.

## Results

### Sample collection

**Figure 1:**
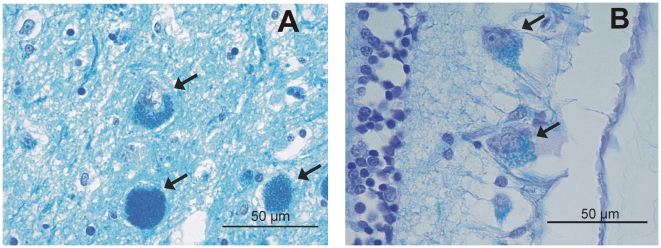
Luxol fast blue (LFB) staining. Of the (A) cerebrum and (B) retina from a NCL-affected Tibetan terrier. Intracytoplasmic LFB positive pigment is indicated with arrows, bar indicates magnification for the micrograph (50 µm).

We collected samples from 24 NCL-affected Tibetan terriers and 1,347 Tibetan terriers showing no clinical signs of NCL. Due to the late-onset of NCL, non-affected Tibetan terriers aged <4–7 years can manifest signs of NCL later in life. All 24 NCL-affected Tibetan terriers showed clinical signs of NCL at an average of six years. Necropsy was performed on seven of the NCL-affected Tibetan terriers. Histological examination of retina, cerebellum, cerebrum and spinal cord revealed gold-brown intracytoplasmic pigment within retinal ganglion cells and neurons, respectively, associated with degeneration and glioses using hematoxylin eosin staining. In addition, cytoplasmic accumulations show a positive signal using periodic acidic-Schiff reaction (PAS) and luxol fast blue staining (LBF) (Figure 1). Brain iron accumulation using Turnbull blue staining could not be found in affected dogs. The phenotype of controls has been assured through reports of the owners and veterinary examinations. All controls had an age of at least 10 years and all their registered offspring was ascertained as NCL-clear. The controls were distantly related to families with NCL-affected dogs using five generation pedigree records. These requirements made it difficult to collect a larger number of controls fulfilling these requirements.

### Mapping the causative genomic region

A genome-wide association analysis was performed for 12 Tibetan-terrier-NCL-cases and 7 Tibetan-terrier-NCL-controls using the 127K canine Affymetrix SNP chip (Affymetrix, Santa Clara, CA, USA). Controls were either not closely related with the controls nor with the cases what caused an unbalanced design. Chromosomal regions with genome-wide significant associations (−log_10_p-values>3.2–3.5) were located on dog chromosomes (CFA) 2 at 81.3–86.2 Mb, on CFA8 at 61 Mb, on CFA12 at 60.2–61.4 Mb, on CFA18 at 48.4–50.3 Mb, on CFA22 at 48 Mb and on CFA37 at 29 Mb using PLINK for association testing with adaptive permutations. We were not able to identify one homozygous haplotype shared among all cases in these associated regions. In order to rule out false positively associated regions and to exclude heterogeneity, we used two different approaches. First, we employed multipoint non-parametric linkage analyses and secondly, we enlarged the number of cases and controls and we tested SNPs from significantly associated regions on CFA2, 8, 12 and 18 in this larger data set. A total of 28 microsatellites (Table S1) covering the associated and their adjacent chromosomal regions were employed in three families including 107 Tibetan terriers (Table S2, Figure S1). Mendelian inheritance and correctness of marker transmission in the pedigrees genotyped was confirmed using Pedstats [15]. A non-parametric multipoint linkage analysis has been employed as this approach does not require assumptions on the mode of inheritance and specification of genetic parameters such as mode of inheritance, penetrance and allele frequencies and so this approach should be useful for traits when the correct mode of inheritance is unknown or assumptions of genetic parameters may lead to ambiguous results or heterogeneity might be of importance. We ruled out linkage for NCL for the chromosomes 8, 18, 22 and 37 (Table S3). Evidence for chromosome-wide significant linkage of the genomic region at 83.22–88.0 Mb on canine chromosome 2 was affirmed. A total of 13 SNPs (Table S4) was genotyped for the significantly associated regions on CFA2, 8, 12 and 18 in 376 Tibetan terriers whereof 24 were NCL-affected and 30 parents or offspring of NCL-affected dogs. These 376 Tibetan terriers also contained the 107 dogs out of the three families for linkage analysis and further 114 Tibetan terriers to increase the number of family members of these three families and in addition, 111 Tibetan terriers belonging to 17 different pedigrees. Genotyping was performed using the Sequenom technology (Sequenom, Hamburg, Germany). A significant association (−log_10_p-value = 6.7) could only be shown for one SNP (BICF2S23719003) located on CFA2 at 83.94 Mb.

Mixed model analysis (MMA) was employed to ensure the associated region on CFA2 and to narrow down the associated region. The advantage of MMA over a simplistic approach without considering any other effects in modelling association can be seen in removing disturbing effects caused by data structure, different levels of relationships among animals and inbreeding. The model included the respective SNP genotypes, sex and inbreeding coefficients as fixed effects and the genomic relationship matrix for the random genetic effect of the animal. Using MMA the NCL-region mapped to 83.71–84.719 Mb on dog chromosome 2 (−log_10_p-value>200; Figure S2).

### Identification and sequence analysis of candidate genes

In the region at 81.17–87.29 Mb on CFA2, we evaluated 14 genes for late-onset NCL in Tibetan terriers (Table S5). For sequence analysis cDNA (Table S6) from two NCL-affected Tibetan terriers and one control dog was used. Polymorphisms detected (Table S7) were confirmed by re-sequencing genomic DNA from four NCL-affected and four NCL-unaffected Tibetan terriers. In the 3′UTR of the canine *MAPK PM20/PM21* gene one SNP (XM_846908:c.766T>C) was detected showing co-segregation with the NCL-phenotype in Tibetan terriers. To evaluate this mutation the same 376 Tibetan terriers employed for association analysis were genotyped for the c.766T>C SNP (Table S8). In this sample the NCL-associated allele C had a frequency of 0.265 and the frequency of T/T genotypes was 0.559. The associations for genotypes and alleles were significant at −log_10_p>15 with χ^2^-values of 194.58 and 94.16. Out of the 24 known NCL-affected dogs, 22 affected could be correctly affirmed and from the 30 NCL-carriers ascertained from the pedigrees, 27 could be correctly identified with the c.766T>C SNP.

### Mutation analysis of *ATP13A2*

**Figure 2:**
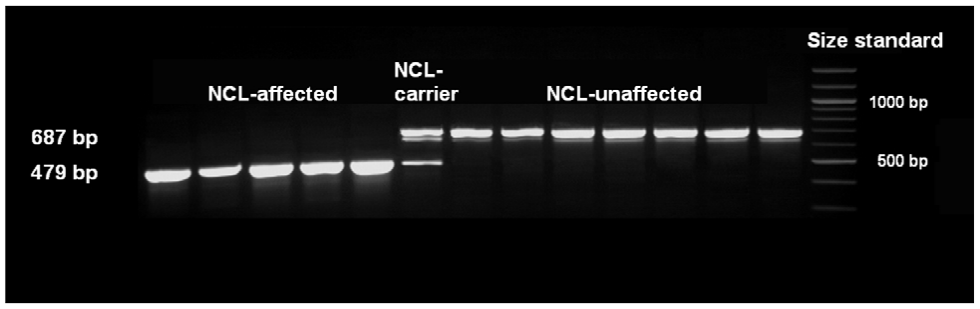
Mutation analysis of exon 16 using cDNA. Primer pairs used were ATP13A2_F7 and ATP13A2_R7. Agarose gel analyses revealed exon 16 skipping for NCL-affected Tibetan terriers.

A candidate gene in close vicinity to the highest peaks of the association analyses is the canine *ATPase type 13A2* (*ATP13A2*) encoding a lysosomal type 5 ATPase in human known to be involved in Kufor-Rakeb-syndrome, a variant of Parkinson disease. This gene is annotated at 84.09–84.11 Mb in Ensemble canine genome reference sequence but not in CanFam 2.1 of NCBI. Using the mRNA (Accession number NM_001141973) of *ATP13A2* and BLAST for canine expressed sequence tags (ESTs), we found five canine ESTs for the *ATP13A2* gene (Accession numbers DN355771, DN374414, DN442497, DN743972, DN905373) and could predict the canine *ATP13A2* gene at 84.09–84.11 Mb on CFA2. The canine *ATP13A2* gene model contains like the human transcript variant ENST00000326735 gene 29 exons with an open reading frame of 3,522 bp coding for a protein with 1,173 amino acids. The Ensemble canine cDNA reference sequence starts with exon 2 and exon 1 is missing. Re-sequencing of the canine *ATP13A2* using cDNA from five NCL-affected and two NCL-unaffected Tibetan terrier confirmed the predicted gene model. Mutation analysis of the amplified sequences revealed exon skipping of exon 16 in all 5 NCL-affected dogs. To verify this mutation exon 16 and its flanking introns were sequenced using genomic DNA from seven NCL-affected and 30 NCL-unaffected Tibetan terrier. In the canine *ATP13A2* gene a single base pair deletion within exon 16 (c.1620delG, Figure S3) was identified that causes skipping of exon 16 (Figure S4, Figure 2) in NCL-affected dogs. Sequencing of the whole introns 15 and 16 revealed no mutations and thus, an intronic mutation responsible for skipping exon 16 was not found. Exon skipping does not shift the open reading frame (ORF) but leads to a protein shortened by 69 amino acids (Figure S5). The loss of amino acids in the canine ATP13A2 protein destroys the signatures of the P-type cation transporting ATPase superfamily and sodium/potassium-transporting ATPase, the E1-E2 ATPases phosphorylation site and a part of the domain of the haloacid dehalogenase-like hydrolase (Figure S5).

### Confirmation of the association in the Tibetan terrier population

The single base pair deletion in exon 16 alters the restriction site of the restriction enzyme BglI. Restriction fragment length polymorphism (RFLP) was developed for screening the Tibetan terrier population. In dogs with the genotype homozygous for G, the 635 base pair (bp) PCR product was cut into two fragments with 322 and 313 bp. In NCL-cases carrying the deletion homozygously, the amplicons were not cut and only the 635 bp product could be observed.

**Figure 3:**
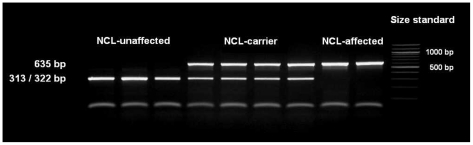
Detection of the *ATP13A2* one base pair deletion. The deletion was genotyped using a restriction fragment length polymorphism on a 4% agarosegel and the restriction enzyme BglI. NCL-affected Tibetan terriers show only one fragment, NCL-carriers show two fragments and NCL-unaffected Tibetan terriers show only one fragment, because the 312 and 323 bp fragments are not separated on the agarosegel.

We screened 168 Tibetan terriers for the c.1620delG using BglI. All genotyped 24 known NCL-cases were homozygous for the deletion in exon 16 and the 35 known NCL-carriers heterozygous for the deletion (Figure 3, Table 1). Screening of the 168 Tibetan terriers revealed ten not yet known NCL-cases and further 35 NCL-carriers. The rest of the Tibetan terriers screened for c.1620delG showed the wild type genotype. Tibetan terriers expected as free for the c.1620delG mutation were homozygous G/G like the dog reference sequence. The frequency of the mutated allele was 0.309. To assure the observed results, 144 dogs from eleven different dog breeds were genotyped for the c.1620delG mutation. All 144 dogs did show the wild type genotype (Table S10).

**Table 1:** Summary of results for the *ATP13A2* c.1620delG mutation in Tibetan terriers.

*ATP13A2* c.1620delG p.A526*	Known NCL- affected dogs (n = 24)	Newly identified as NCL-affected	Known NCL- carriers (n = 35)	Newly identified NCL- carriers	Controls (n = 18)	Newly identified as NCL- unaffected
G/G	-	-	-	-	18	109
G/delG	-	-	35	97	-	-
delG/delG	24	21	-	-	-	-

## Discussion

A 1-bp deletion within exon 16 of *ATP13A2* (c.1620delG) was identified as responsible for NCL in the Tibetan terrier. All NCL-cases reported in Tibetan terriers were homozygous for this mutation and therefore heterogeneity seems unlikely for this disease in Tibetan terriers. This c.1620delG mutation causes an alternative splicing of exon 16 but not a frameshift mutation with a premature termination codon as previously supposed [14]. As a result of the in-frame loss of exon 16, the ATP13A2 protein is shortened by 69 amino acids. Therefore, all NCL-affected Tibetan terriers in the present study can synthesize this shortened ATP13A2 protein. In humans, all three isoforms do not lack exon 16. This new insight on the structure of the mutated protein may explain why Tibetan terriers express only mild neurodegenerative symptoms and the onset of the disease is late in life.

Exonic substitutions often create or eliminate short elements that inhibit or activate exon inclusion or splice-site selection [16]–[18]. These splicing silencers or enhancers are present both in exons (ESSs, ESEs) and introns (ISSs, ISEs) and have been derived by computational and/or experimental approaches [19]–[20]. Searching the mutated canine *ATP13A2* sequences using Human Splicing Finder, version 2.4 (http://www.umd.be/HSF/) [19] we could affirm that the 206 bp-exon 16 is no longer recognized by splicing sites because the 1-bp deletion is responsible for a broken exonic splicing enhancer motif sequence. However, the donor site at the exon 15 boundary and the acceptor site at the exon 17 boundary are still recognized. Thus, we assume that the splicing process for exon 16 fails since the c.1620delG mutation destroys the ESE motif (“CCGCCTG” at cDNA positions 1614–1620) within exon 16.

The *ATP13A2* gene encodes a member of the P5 subfamily of ATPases which transports inorganic cations as well as other substrates. Mutations in this gene have been identified in Kufor-Rakeb syndrome (KRS) patients, a familial form of Parkinson disease (PARK9). One of the mutation studies described a 22 bp-duplication mutation in exon 16 of the human *ATP13A2* gene [21]. A milder form of KRS than that reported for frameshift or truncating mutations was caused by a G504R missense mutation in exon 15 of the *ATP13A2* gene, affecting the cytosolic loop close to the predicted catalytic phosphorylation site [22]. The clinical signs of PARK9 of this patient including aggressive behaviors resemble some of the signs of NCL seen in affected Tibetan terriers. Therefore, NCL in Tibetan terriers might be a mild form of KRS with Parkinson's disease-like symptoms since a truncating mutation was not present in Tibetan terriers. The protein-shortening mutation found in Tibetan terriers seems to be the reason why only a partial overlap of disease symptoms among KRS patients due to a truncating mutation and NCL-affected Tibetan terriers is observed. A wide intra-familial clinical variability of PARK9-linked disease seems to exist in humans. In agreement with the NCL-affected Tibetan terriers, there were also cases in man with no evidence for brain iron accumulation [23]. Knock-down of the *PARK9* orthologue in *C. elegans* enhances α-synuclein (α–syn) misfolding [24]. This *PARK9* functional relationship with the α–syn pathobiology could be confirmed in rat primary midbrain neuron cultures. On the other hand, yeast *PARK9* helps to protect cells from manganese toxicity [24].

To assure that the detected deletion within exon 16 is causative for NCL in Tibetan terriers, all NCL-affected and NCL-carrier dogs were genotyped and in addition, random samples of 144 dogs from eleven different dog breeds were tested for the presence of the c.1620delG mutation. All individuals of these nine different dog breeds were homozygous for the wild type sequence. The c.1620delG mutation of the *ATP13A2* gene was not yet reported as responsible for exon skipping and can therefore be regarded as novel in this regard.

In conclusion, we identified the causal mutation for canine late-onset NCL in the Tibetan terrier. This mutation (c.1620delG) is located within exon 16 of *ATP13A2* and leads to a shortened protein through an alternative splicing process for exon 16 due to a broken ESE motif. The pathobiology may be mediated through the connection of *ATP13A2* to the α–syn network. Tibetan terriers carrying the susceptible genotype for NCL may be a valuable model for unraveling the pathobiology of a PARK9-linked disease and testing the role of manganese toxicity in synucleinopathies. Knowledge of the causative mutation for late-onset NCL in Tibetan terriers allows genetic testing of these dogs to avoid matings of carrier animals. So the breeders are able to eliminate this disease in their breeding lines.

## Materials and Methods

### Ethics statement

All animal work has been conducted according to the national and international guidelines for animal welfare. All blood-sampling of NCL-affected dogs was done in veterinary clinics for small animals during the routine of diagnosis of NCL. The blood samples of unaffected dogs were provided from veterinarians in veterinary clinics. Samples for RNA isolation were taken in veterinary clinics for small animals after euthanasia.

### Sample collection and processing

EDTA-blood samples from affected and control dogs were collected from 1,371 Tibetan terriers. Thereof 24 Tibetan terriers were NCL-affected, 35 were NCL-carriers because they were parents or offspring of NCL-affected dogs and the other 1,312 Tibetan terriers had a clear or unknown phenotype. Except of one NCL-affected Tibetan terrier from Switzerland all NCL-affected Tibetan terriers were from Germany. Within the known NCL-carriers, three dogs were from Denmark, two from Switzerland and each one from Finland and USA.

### DNA extraction

Genomic DNA was extracted from EDTA blood samples through a standard ethanol fractionation with concentrated sodiumchloride (6 M NaCl) and sodium dodecyl sulphate (10% SDS). Concentration of extracted DNA was determined using the Nanodrop ND-1000 (Peqlab Biotechnology, Erlangen, Germany). DNA concentration of samples for SNP chip analysis was adjusted to 50–70 ng/µl.

### RNA extraction and cDNA synthesis

For cDNA analysis, biopsies from conjunctiva and cerebrum of five NCL-affected dogs during the pathological examination were taken. These samples were taken 15–30 minutes after the dogs were euthanised.

Tissue samples were conserved using RNA-later solution (Qiagen, Hilden, Germany). The RNA was extracted from the cerebrum tissues using the RNeasy Lipid Tissue Mini Kit (Qiagen) and transcribed into cDNA using SuperScript III Reverse Transcriptase (Invitrogen, Karlsruhe, Germany).

### Statistical analysis

Genome-wide association analyses were performed using the 127K canine Affymetrix SNP chip (Affymetrix, Santa Clara, CA, USA). DNA from 12 Tibetan-terrier-NCL-cases and 7 Tibetan-terrier-NCL-controls were used for a genome-wide association analysis. The phenotype of controls has been assured through reports of the owners and veterinary examinations. All controls had an age of at least 10 years and all their registered offspring was ascertained as NCL clear. The controls were preferably unrelated to families with NCL affected dogs using five generation pedigree records.

Quality criteria were minor allele frequencies (MAF) >2% and SNP genotyping rates >95% for the genome-wide association for NCL of the resulting 111,525 SNPs from the canine 125K SNP chip were performed using PLINK, version 1.07 (http://pngu.mgh.harvard.edu/purcell/plink/) [25] and Tassel, version 2.1 [26]. Genome-wide significance was ascertained through adaptive permutation testing using a maximum of 5,000,000 permutations.

Non-parametric multipoint linkage (NPL) analysis was performed for the NCL-affected Tibetan terriers and their relatives from three different families using MERLIN 1.1.2 [27]. Linkage among NCL and microsatellites was estimated using the proportion of alleles identical by descent (IBD) for affected animals. The NPL statistics Zmean and the LOD (logarithm of the odds) scores were employed for detection of allele sharing among affected family members. The maximum (minimum) achievable Zmean and LOD score were 6.83 (−2.10) and 2.60 (−0.31) indicating enough power to detect chromosome-wide significant linkage. In the case of no linkage, Zmean approaches the minimum achievable value due to an equal distribution of alleles among affected relatives. When linkage is present under the alternative hypothesis, the proportion of alleles IBD significantly deviates from the expected IBD proportions of the null hypothesis. We employed multipoint analyses in order to use marker information from the whole chromosome through linked informative markers and to increase power of linkage analysis.

### Sequencing and genotyping

For cDNA analysis of genes in the associated genomic region annotated canine mRNA sequences were used. To assure the annotation we searched the dog expressed sequence tag (EST) archive (http://www.ncbi.nlm.nih.gov/genome/seq/CfaBlast.html) for ESTs by cross-species BLAST searches with the corresponding human reference mRNA sequences.

For PCR primer design the PRIMER3 software (http://frodo.wi.mit.edu/cgi-bin/primer3/primer3_www.cgi) were used. Primer pairs used for cDNA amplification of the 14 positional candidate genes are given in Tables S6 and S9. The PCR reactions were performed in a total volume of 50 µl containing 10 ng of genomic DNA as template, 10 pmol of each primer and 1 U Taq polymerase (MP Biomedicals, Eschwege, Germany). Thermocycling was carried out under the following conditions: initial denaturation at 94°C for 4 min was followed by 35 cycles of 94°C for 30 s, 58–61°C for 30 s, 72°C for 1∶20 min and a final step with 72°C for 5 min before cooling at 4°C for 10 min. Primer pairs for amplification the genomic DNA of canine *ATP13A2* exon 16 were as follows: ATP13A2_F 5′-GACCTGCCGTAGGGTGAAG-3′ and ATP13A2_R5′-AAGCTTCCTTCCTGGGCTAC-3′. Amplicons were directly sequenced using an ABI 3700 capillary sequencer (Life Technologies, Darmstadt, Germany). Afterwards sequence data were analyzed using Sequencher software version 4.7 (GeneCodes, Ann Arbor, MI, USA).

### Microsatellite development and analysis

We genotyped 28 microsatellites on CFA2, 8, 18, 22 and 37. We used 16 markers of the canine minimal screening set 2 [28] and 12 newly developed markers. Microsatellite motifs were identified in the assembled dog sequences (dog genome assembly 2.1) using BLAST http://www.ncbi.nlm.nuh.gov/entrez/query.fcgi). The criterion for markers to be included in this set was more than 15 repeats of di- tri- tetra- or pentanucleotide motifs, respectively. Prior to primer design using PRIMER3 repetitive sequences were masked employing repeatmasker (http://www.repeatmasker.org). The average marker distance was about 0.5 Mb at 81.5–88.0 Mb on CFA2.

The PCR was carried out with an initial denaturing for 4 min at 94°C followed by 38 cycles with denaturing at 94°C for 30 sec, optimum primer annealing temperature (Table S1) for 30 sec and elongation at 72°C for 45 sec. All PCR reactions were performed in 12.0-µl reactions using 10 pmol of each primer, 0.2 µl dNTPs (100 µM) and 0.1 µl *Taq*-DNA-Polymerase (5 U/µl) (Q-Biogen, Heidelberg, Germany) in the reaction buffer supplied by the manufacturer for 2 µl template DNA. The forward primers were labelled fluorescently with IRD700 or IRD800. For the analysis of the marker genotypes, PCR products were size-fractionated by gel electrophoresis on an automated sequencer (LI-COR, Lincoln, NE, USA) using 4% polyacrylamide denaturing gels (Rotiphorese Gel40, Carl Roth, Karlsruhe). Allele sizes were determined using an IRD700- and IRD800-labelled DNA ladder. Genotypes were assigned by visual examination.

### Pathological-histological examination

Formalin-fixed, paraffin-embedded tissue samples of the retina, cerebellum, cerebrum and spinal cord were examined by histology. Briefly, 3 µm-thick sections were cut on a microtome and mounted on glass slides. Subsequently slides were stained with hemotoxylin and eosin (HE), periodic acid-Schiff reaction (PAS), Turnbull blue and luxol fast blue staining (LFB).

## Supporting Information

Figure S1 Pedigree structure of the three Tibetan terrier families used for linkage analysis. (A) Family 1, (B) family 2 and (C) family 3. Tibetan terriers in the families marked with a “g” were genotyped for linkage analysis and Tibetan terriers marked with “chip” were also used for the 127K canine Affymetrix SNP chip analysis. Some females were mated to different sires causing mating loops.
(DOC)

Figure S2 Manhattan-plot of the −log_10_ P-values for the genome-wide association analysis of late-onset neuronal ceroid lipofuscinosis (NCL) in Tibetan terriers from a mixed model analysis using TASSEL, version 1.07. The highest −log_10_ p-values (>200) were obtained for dog chromosome 2 at 83.7–84.7 Mb.
(DOC)

Figure S3 Causative mutation for late-onset NCL in Tibetan terriers. Chromatograms of genomic sequences of *ATP13A2* exon 16 from each one NCL-affected, NCL-carrier, NCL-free Tibetan terrier and the dog reference sequence. The 1-bp deletion at base pair 96 of exon 16 marked by a red rectangle and 15 bp up- and 15 bp downstream sequence are shown in the lower part of the figure.
(DOC)

Figure S4 Exon splicing patterns of transcripts from normal and mutant *ATP13A2* alleles. cDNA from the normal allele contains exon 16, the mutant allele skips exon 16 and exon 15 is spliced to exon 17 splice acceptor site. Skipping of exon 16 leads to a loss of 69 amino acids. The open reading frame remained unchanged through the loss of exon 16.
(DOC)

Figure S5 CLUSTAL 2.0.12 multiple protein alignment. The human ATP13A2 isoform 1 protein has 1175 amino acids, the normal canine ATP13A2 protein 1173 amino acids and the canine NCL-associated ATP13A2 protein is shortened by 69 amino acids.
(DOC)

Table S1 Microsatellite markers for fine mapping the genomic region on canine chromosome 2, 8, 18, 22 and 37. The primer pairs used are given with their position in mega bases (Mb), annealing temperature (AT), heterozygosity (HET) and their polymorphism information content (PIC). HET and PIC were calculated for 107 Tibetan terriers.
(DOC)

Table S2 Tibetan terrier families used for linkage analysis. For linkage analysis 20 NCL-affected and 87 NCL-unaffected Tibetan terriers were genotyped in three families enclosing a total of 107 Tibetan terriers.
(DOC)

Table S3 Chromosome-wide error probabilities (p_Zmean_, p_LOD_) for multipoint non-parametric linkage analysis in 107 Tibetan terriers for the NCL-phenotype using 28 microsatellites.
(DOC)

Table S4 Summary results for the SNPs used for association analysis. SNP accession numbers, positions and alleles genotyped for 376 Tibetan terriers with their minor allele frequencies (MAF) and P-values for genotypic association with NCL.
(DOC)

Table S5 Candidate genes evaluated for late-onset NCL in Tibetan terriers. Gene name, annotation by NCBI or Ensembl, position in mega bases (Mb) and involvement in disease/function of genes in human.
(DOC)

Table S6 Primer pairs for cDNA amplification and the genomic regions of the 14 canine candidate genes. The primer pairs are given with their sequences, annealing temperature (AT), product size in base pairs (bp) and the target position.
(DOC)

Table S7 Single nucleotide polymorphisms detected in coding and untranslated sequences of the evaluated genes for NCL in Tibetan terriers. SNP nomenclature, position and possible amino acid exchange are given.
(DOC)

Table S8 Genotyping 376 Tibetan terriers for the canine *MAPK PM20/PM21* c.766T>C SNP.
(DOC)

Table S9 Primer pairs for cDNA amplification of the canine *ATP13A2* gene and its genomic DNA surrounding exon 16. The primer pairs are given with their sequences, annealing temperature (AT), product size in base pairs (bp) and the target position.
(DOC)

Table S10 Dog breeds other than Tibetan terrier genotyped for the *ATP13A2* c.1620delG mutation.
(DOC)
