# Supplementary figures and images for: A One Base Pair Deletion in the Canine ATP13A2 Gene Causes Exon Skipping and Late-Onset Neuronal Ceroid Lipofuscinosis in the Tibetan Terrier
Source: PLoS Genet. 2011 Oct 13;7(10):e1002304. doi: 10.1371/journal.pgen.1002304 (PMC3192819; doi:10.1371/journal.pgen.1002304)

**
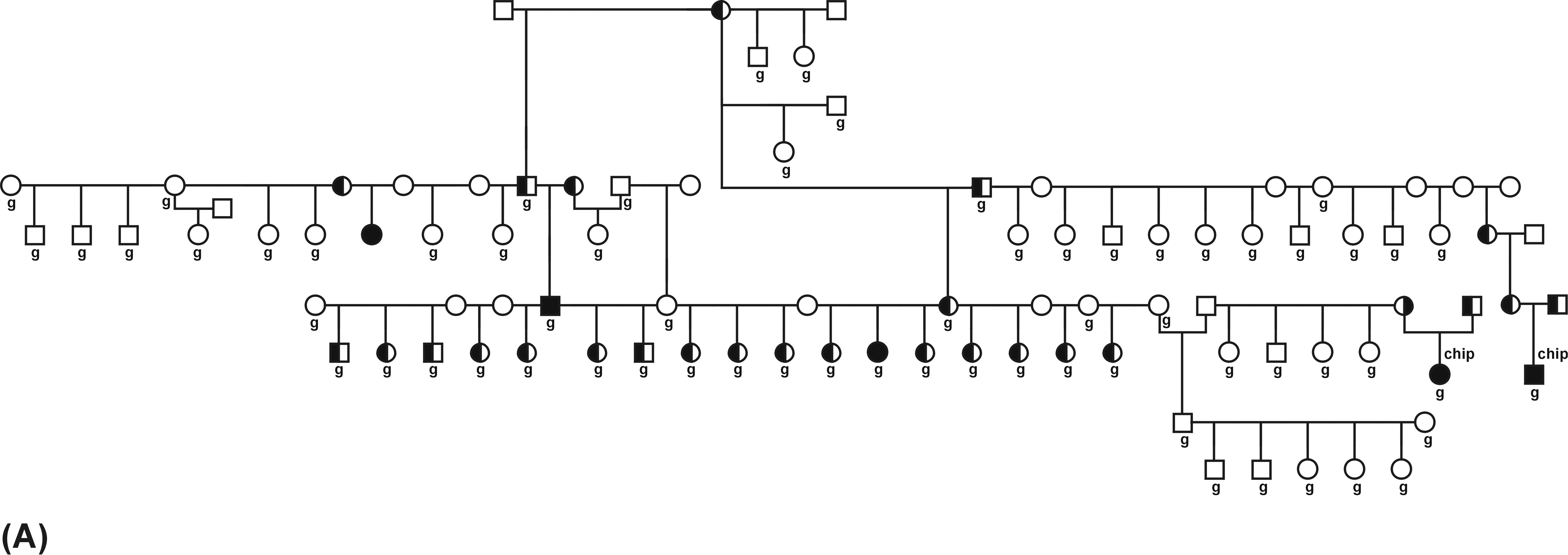
**

**
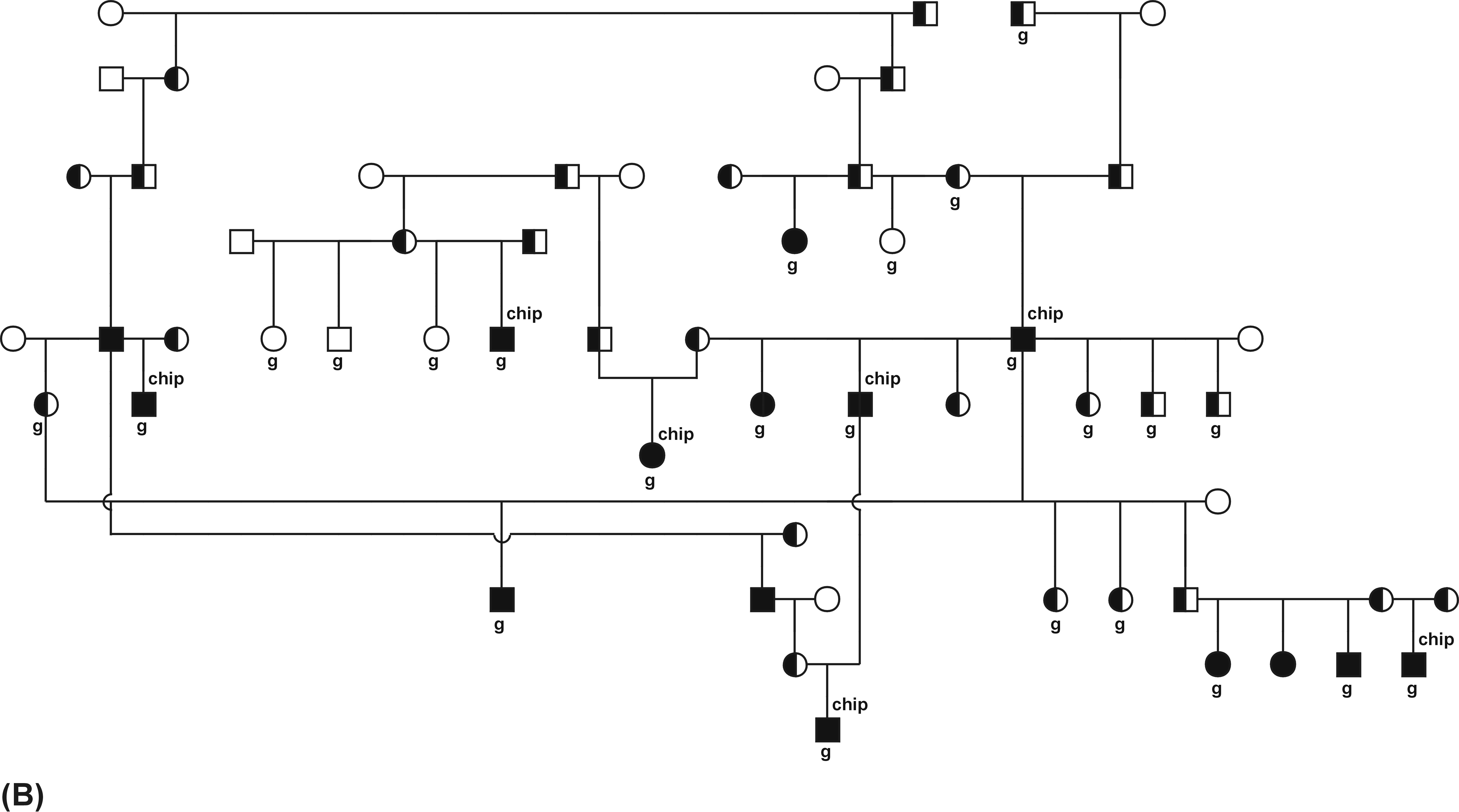
**

**
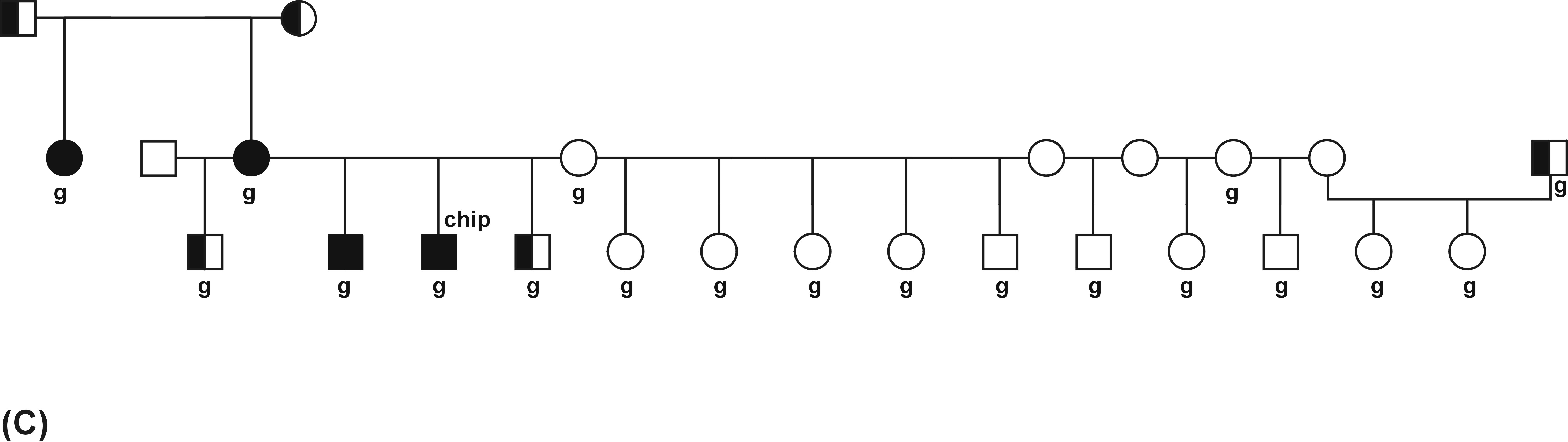
**

Supplement: Figure S1 — Pedigree structure of the three Tibetan terrier families used for linkage analysis. (A) Family 1, (B) family 2 and (C) family 3. Tibetan terriers in the families marked with a “g” were genotyped for linkage analysis and Tibetan terriers marked with “chip” were also used for the 127K canine Affymetrix SNP chip analysis. Some females were mated to different sires causing mating loops. (DOC) [file pgen.1002304.s001.doc]

**
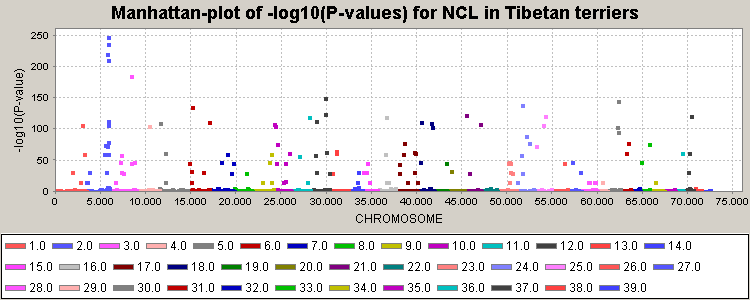
**

Supplement: Figure S2 — Manhattan-plot of the −log10 P-values for the genome-wide association analysis of late-onset neuronal ceroid lipofuscinosis (NCL) in Tibetan terriers from a mixed model analysis using TASSEL, version 1.07. The highest −log10 p-values (>200) were obtained for dog chromosome 2 at 83.7–84.7 Mb. (DOC) [file pgen.1002304.s002.doc]

**
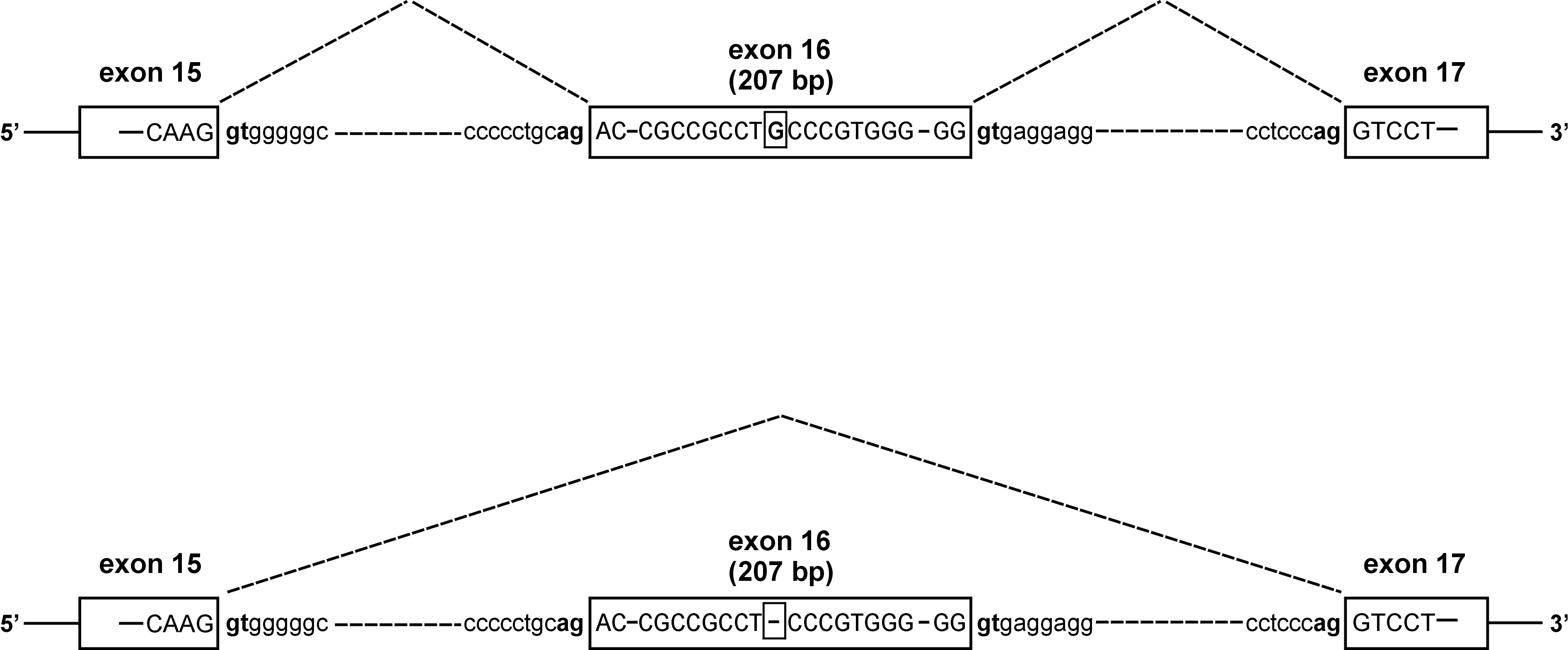
**

Supplement: Figure S4 — Exon splicing patterns of transcripts from normal and mutant ATP13A2 alleles. cDNA from the normal allele contains exon 16, the mutant allele skips exon 16 and exon 15 is spliced to exon 17 splice acceptor site. Skipping of exon 16 leads to a loss of 69 amino acids. The open reading frame remained unchanged through the loss of exon 16. (DOC) [file pgen.1002304.s004.doc]
