# Supplementary material for: A One Base Pair Deletion in the Canine ATP13A2 Gene Causes Exon Skipping and Late-Onset Neuronal Ceroid Lipofuscinosis in the Tibetan Terrier
Source: PLoS Genet. 2011 Oct 13;7(10):e1002304. doi: 10.1371/journal.pgen.1002304 (PMC3192819; doi:10.1371/journal.pgen.1002304)
Supplement: Figure S5 — CLUSTAL 2.0.12 multiple protein alignment. The human ATP13A2 isoform 1 protein has 1175 amino acids, the normal canine ATP13A2 protein 1173 amino acids and the canine NCL-associated ATP13A2 protein is shortened by 69 amino acids. (DOC) [file pgen.1002304.s005.doc]

canine MSADSSPLVGSTPAGYGTLTIETSVDPLSSSVSSVRLSGYCGSPWRVIGYHIVVWMMAGI 60

short MSADSSPLVGSTPAGYGTLTIETSVDPLSSSVSSVRLSGYCGSPWRVIGYHIVVWMMAGI 60

human MSADSSPLVGSTPTGYGTLTIGTSIDPLSSSVSSVRLSGYCGSPWRVIGYHVVVWMMAGI 60

*************:******* **:**************************:********

canine PLLLFRWKPVWGVRLRLRPCNLAHAETLVIEIRDKEDNSWQLYTVQVQTEAIGEGSLELP 120

short PLLLFRWKPVWGVRLRLRPCNLAHAETLVIEIRDKEDNSWQLYTVQVQTEAIGEGSLELP 120

human PLLLFRWKPLWGVRLRLRPCNLAHAETLVIEIRDKEDSSWQLFTVQVQTEAIGEGSLEPS 120

*********:***************************.****:*************** .

canine ARGQAEDGRSQAAVGAVPEGAWKDTAQFCKNEEAR-MLRYYLFRGQRYVWIESQQAFCQA 179

short ARGQAEDGRSQAAVGAVPEGAWKDTAQFCKNEEAR-MLRYYLFRGQRYVWIESQQAFCQA 179

human PQSQAEDGRSQAAVGAVPEGAWKDTAQLHKSEEAKRVLRYYLFQGQRYIWIETQQAFYQV 180

.:.************************: *.***: :******:****:***:**** *.

canine SLLDNGRTCEDVHRSCSGLSLQDQAVRKTIYGPNVISVPVKSYPQLLVDEALNPYYGFQA 239

short SLLDNGRTCEDVHRSCSGLSLQDQAVRKTIYGPNVISVPVKSYPQLLVDEALNPYYGFQA 239

human SLLDHGRSCDDVHRSRHGLSLQDQMVRKAIYGPNVISIPVKSYPQLLVDEALNPYYGFQA 240

****:**:*:***** ******* ***:********:**********************

canine FSIGLWLADRYYSYALCIFLISTASICLSLYKTRKQSQTLRDMVQLSARVCVCRPGGEEE 299

short FSIGLWLADRYYSYALCIFLISTASICLSLYKTRKQSQTLRDMVQLSARVCVCRPGGEEE 299

human FSIALWLADHYYWYALCIFLISSISICLSLYKTRKQSQTLRDMVKLSMRVCVCRPGGEEE 300

***.*****:** *********: ********************:** ************

canine WVDSSELVPGDCLVLPREGGLVPCDAALVAGECVVNESSLTGESVPVLKTALPEGPVSYC 359

short WVDSSELVPGDCLVLPREGGLVPCDAALVAGECVVNESSLTGESVPVLKTALPEGPVSYC 359

human WVDSSELVPGDCLVLPQEGGLMPCDAALVAGECMVNESSLTGESIPVLKTALPEGLGPYC 360

****************:****:***********:**********:********** .**

canine PETHRRHTLFCGTLVLQARAFVGPHVLAVVTQTGFCTAKGGLVSSILHPRPIDFKFYKHS 419

short PETHRRHTLFCGTLVLQARAFVGPHVLAVVTQTGFCTAKGGLVSSILHPRPIDFKFYKHS 419

human AETHRRHTLFCGTLILQARAYVGPHVLAVVTRTGFCTAKGGLVSSILHPRPINFKFYKHS 420

.*************:*****:**********:********************:*******

canine MKFVAALSVLALLGTVYSIFILHRNRVPLNEIVIRALDLVTVVVPPALPAAMTVCTLYAQ 479

short MKFVAALSVLALLGTVYSIFILHRNRVPLNEIVIRALDLVTVVVPPALPAAMTVCTLYAQ 479

human MKFVAALSVLALLGTIYSIFILYRNRVPLNEIVIRALDLVTVVVPPALPAAMTVCTLYAQ 480

***************:******:*************************************

canine SRLRSQGIFCIHPLRINLGGKLQLVCFDKTGTLTEDGLDVMGVVPLKGQAFLPLVPEPRR 539

short SRLRSQGIFCIHPLRINLGGKLQLVCFDK------------------------------- 508

human SRLRRQGIFCIHPLRINLGGKLQLVCFDKTGTLTEDGLDVMGVVPLKGQAFLPLVPEPRR 540

**** ************************

canine LPVGPLLRALATCHALSRLQDTPVGDPMDLKMVESTGWVLEEGPAADSAFGDQVLAVMRP 599

short --------------------------------------VLEEGPAADSAFGDQVLAVMRP 530

human LPVGPLLRALATCHALSRLQDTPVGDPMDLKMVESTGWVLEEEPAADSAFGTQVLAVMRP 600

canine PLQEAQLQGREEPPVPVSILNRFPFSSALQRMNVVVAWPGAAQPEAYVKGSPELVAGLCN 659

short PLQEAQLQGREEPPVPVSILNRFPFSSALQRMNVVVAWPGAAQPEAYVKGSPELVAGLCN 590

human PLWEPQLQAMEEPPVPVSVLHRFPFSSALQRMSVVVAWPGATQPEAYVKGSPELVAGLCN 660

canine PETVPADFAQMLQSYTAAGYRVVALASKPLPIVPSLEAAQQLSRDAVERELTLLGLLVMR 719

short PETVPADFAQMLQSYTAAGYRVVALASKPLPIVPSLEAAQQLSRDAVERELTLLGLLVMR 650

human PETVPTDFAQMLQSYTAAGYRVVALASKPLPTVPSLEAAQQLTRDTVEGDLSLLGLLVMR 720

canine NLLKPQTTPVIQALRRTRIRTVMVTGDNLQTAVTVAQGCGMVGPQERLVIIHATPPERGQ 779

short NLLKPQTTPVIQALRRTRIRTVMVTGDNLQTAVTVAQGCGMVGPQERLVIIHATPPERGQ 710

human NLLKPQTTPVIQALRRTRIRAVMVTGDNLQTAVTVARGCGMVAPQEHLIIVHATHPERGQ 780

canine PASLELLPLESPAAVNGAKDPDQAASYTMEPDPRSSHLALSGSTFGVLMKHFPKLLPKIL 839

short PASLELLPLESPAAVNGAKDPDQAASYTMEPDPRSSHLALSGSTFGVLMKHFPKLLPKIL 770

human PASLEFLPMESPTAVNGVKDPDQAASYTVEPDPRSRHLALSGPTFGIIVKHFPKLLPKVL 840

canine VQGTVFARMAPEQKTELVCELQKLQYCVGMCGDGAHDSRALKAANLGISLSQAEASVVSP 899

short VQGTVFARMAPEQKTELVCELQKLQYCVGMCGDGAHDSRALKAANLGISLSQAEASVVSP 830

human VQGTVFARMAPEQKTELVCELQKLQYCVGMCGDGANDCGALKAADVGISLSQAEASVVSP 900

canine FTSSTACIECVPRVIREGRCSLDTSFSVFKYMALYSLTQFISVLILYTINTNLGDVQFLA 959

short FTSSTACIECVPRVIREGRCSLDTSFSVFKYMALYSLTQFISVLILYTINTNLGDVQFLA 890

human FTSSMASIECVPMVIREGRCSLDTSFSVFKYMALYSLTQFISVLILYTINTNLGDLQFLA 960

canine IDLVITTTVAVLMSRTGPALALGRARPPGALLSVPVLSSLLLHVVLVAGVQLGGYFLTVA 1019

short IDLVITTTVAVLMSRTGPALALGRARPPGALLSVPVLSSLLLHVVLVAGVQLGGYFLTVA 950

human IDLVITTTVAVLMSRTGPALVLGRVRPPGALLSVPVLSSLLLQMVLVTGVQLGGYFLTLA 1020

canine QPWFVPLNKTVPAPDNLPNYENTVVFSLSGFQYLILAAAVSKGAPFRRPLYTNVPFLLAL 1079

short QPWFVPLNKTVPAPDNLPNYENTVVFSLSGFQYLILAAAVSKGAPFRRPLYTNVPFLLAL 1010

human QPWFVPLNRTVAAPDNLPNYENTVVFSLSSFQYLILAAAVSKGAPFRRPLYTNVPFLVAL 1080

canine ALLGSILAGLLLVPGLLQGPLALRNIADTCFKLLLLGLVAFNFVAAFVLESVLDQCLPAC 1139

short ALLGSILAGLLLVPGLLQGPLALRNIADTCFKLLLLGLVAFNFVAAFVLESVLDQCLPAC 1070

human ALLSSVLVGLVLVPGLLQGPLALRNITDTGFKLLLLGLVTLNFVGAFMLESVLDQCLPAC 1140

canine LRRLRPKKASKKRFKQLERELAEQPWPPPTGPMR- 1173

short LRRLRPKKASKKRFKQLERELAEQPWPPPTGPMR- 1104

human LRRLRPKRASKKRFKQLERELAEQPWPPLPAGPLR 1175
