# Supplementary material for: A One Base Pair Deletion in the Canine ATP13A2 Gene Causes Exon Skipping and Late-Onset Neuronal Ceroid Lipofuscinosis in the Tibetan Terrier
Source: PLoS Genet. 2011 Oct 13;7(10):e1002304. doi: 10.1371/journal.pgen.1002304 (PMC3192819; doi:10.1371/journal.pgen.1002304)
Supplement: Table S1 — Microsatellite markers for fine mapping the genomic region on canine chromosome 2, 8, 18, 22 and 37. The primer pairs used are given with their position in mega bases (Mb), annealing temperature (AT), heterozygosity (HET) and their polymorphism information content (PIC). HET and PIC were calculated for 107 Tibetan terriers. (DOC) [file pgen.1002304.s006.doc]

| CFA | Marker | Primer sequence (5’-3’) | Mb | AT | Number of alleles | Size | HET | PIC |
| --- | --- | --- | --- | --- | --- | --- | --- | --- |
|  |  |  |  | (°C) | (n) | in bp |  |  |
| 2 | 2_81.88_F7 | TCAGATTTCAGCAAGCCAAG | 81.88 | 59 | 5 | 238-252 | 66.7 | 72.3 |
|  | 2_81.88_R | GAGAGCAACTCTGTCCCAGG |  |  |  |  |  |  |
| 2 | 2_82.45_F7 | AAGCAGTGAGTTCCCCATTAC | 82.45 | 58 | 6 | 151-181 | 59.7 | 66.6 |
|  | 2_82.45_R | TGGAAACCAGGATCATCTTAAC |  |  |  |  |  |  |
| 2 | 2_82.86_F8 | TCCCAGGATGCATGTGTTAG | 82.26 | 59 | 5 | 286-300 | 43.2 | 46.1 |
|  | 2_82.86_R | AGACGAGAGTCAGCAAGAGAGA |  |  |  |  |  |  |
| 2 | 2_83.22_F7 | TGGGTTCCCTAGTCTCAGGTC | 83.22 | 60 | 3 | 312-328 | 31.5 | 30.6 |
|  | 2_83.22_R | CACTCCATGAAATCACCAAGG |  |  |  |  |  |  |
| 2 | 2_83.77_F8 | CTGCTCTCTTGGAAACCGTC | 83.77 | 60 | 8 | 175-203 | 68.3 | 70.7 |
|  | 2_83.77_R | CATTCTCCTCTTTGCCTTGC |  |  |  |  |  |  |
| 2 | 2_84.19_F7 | CAGACTCCTCTGAGATTTGGAC | 84.19 | 58 | 4 | 177-187 | 47.2 | 55.8 |
|  | 2_84.19_R | GTCCCCAAGGCTTTGAAC |  |  |  |  |  |  |
| 2 | 2_84.23_F7 | AAAACCATCTGAGATTACTTGAGC | 84.23 | 58 | 4 | 284-296 | 47.1 | 62.1 |
|  | 2_84.23_R | CAGCTAATGAAACCCATTGC |  |  |  |  |  |  |
| 2 | 2_85.05_F8 | GACTTCAGTGGCTGATGGAG | 85.05 | 58 | 12 | 190-290 | 67.2 | 73.9 |
|  | 2_85.05_R | TTAGGAAACCCTGCTAGTTCC |  |  |  |  |  |  |
| 2 | 2_85.95_F7 | GAGGGTTGCTGTTGGTTTTC | 85.95 | 60 | 11 | 330-506 | 78.6 | 86.6 |
|  | 2_85.95_R | TATGTGATCGCCATTCAAGG |  |  |  |  |  |  |
| 2 | 2_86.44_F7 | CTTGATCTTGGGATCGCATT | 86.44 | 60 | 10 | 185-285 | 51.6 | 53.7 |
|  | 2_86.44_R | TGGACCTCCTTTGTTTGGTC |  |  |  |  |  |  |
| 2 | 2_87.24_F7 | GCCAGCCCGAAAAAGTCTAT | 87.24 | 59 | 10 | 195-247 | 50.2 | 56.4 |
|  | 2_87.24_R | CACAAGAAATTTTTACTTGGAGTCA |  |  |  |  |  |  |
| 2 | 2_88.00_F8 | CAGCCCCAACCCTAGGTC | 88.0 | 59 | 4 | 145-160 | 21.1 | 24.7 |
|  | 2_88.00_R | TTGCCAGTATCAACACTTCG |  |  |  |  |  |  |
| 2 | AHT111_F | CCATACCCAGGATAGTTGAT | 78.7 | 58 | 3 | 75-85 | 72.8 | 53.0 |
|  | AHT111_R | CCATCCTGAGGCTAGCTGTG |  |  |  |  |  |  |
| 2 | FH2062_F | GGCTTCTGGAGACAGGCAT | 81.9 | 58 | 2 | 132-136 | 52.0 | 36.4 |
|  | FH2062_R | CAGAACGCTGTCTAGCCCT |  |  |  |  |  |  |
| 8 | FH3218_F | CACTCAGCATGGAGTTTGC | 20.9 | 58 | 10 | 205-317 | 71.9 | 70.8 |
|  | FH3218_R | CTCATGTTCACTCATTGAATCC |  |  |  |  |  |  |
| 8 | FH4003_F | TGTAAAGAAGATATGATCCGGG | 29.2 | 62 | 4 | 351-363 | 44.7 | 46.8 |
|  | FH4003_R | CTTTCAATCTCTCTCTTGCTGG |  |  |  |  |  |  |
| 8 | C08.410_F | GAGGAAAACCAAGTGATTTTGG | 34.2 | 58 | 6 | 100-128 | 71.2 | 65.9 |
|  | C08.410_R | ACCTGCAAGTGACCCTCTCT |  |  |  |  |  |  |
| 8 | C08.618_F | CAACCCAGGGTGGAAGC | 68.5 | 56 | 4 | 194-202 | 51.5 | 43.9 |
|  | C08.618_R | TAGCAAGAAAATGTGCCCA |  |  |  |  |  |  |
| 18 | REN248C19_F | TGACTGTGGCAAGCAAGAAC | 44.6 | 60 | 3 | 319-327 | 9.1 | 8.6 |
|  | REN248C19_R | GGCAAAGAAAGATGGACTGG |  |  |  |  |  |  |
| 18 | REN47J11_F | TCTCCTCGCGTGTTTCTG | 45.3 | 55 | 5 | 165-179 | 41.6 | 39.4 |
|  | REN47J11_R | GGGGACACTCAGAAGGACG |  |  |  |  |  |  |
| 18 | REN50L03_F | TTCTTGGGTGTGATAATAGTG | 47.0 | 54 | 4 | 263-271 | 45.5 | 40.2 |
|  | REN50L03_R | TGCACCTTTCACCTACATT |  |  |  |  |  |  |
| 18 | FH2429_F | GATCCACTTTGAATTGATTTTTG | 53.8 | 54 | 9 | 171-191 | 57.4 | 54.7 |
|  | FH2429_R | TTCAGCAAATGGTTCTGGAA |  |  |  |  |  |  |
| 22 | REN49F22_F | GGGGCTCTGTTATTAGGTG | 3.9 | 49 | 3 | 149-157 | 38.4 | 33.6 |
|  | REN49F22_R | TCATAAGGCAAAGAAAACC |  |  |  |  |  |  |
| 22 | REN68D20_F | GCCGCTTTTCAGGTGTC | 5.5 | 52 | 4 | 225-277 | 37.0 | 32.5 |
|  | REN68D20_R | TCAAATGGGGAAAGAAAAACT |  |  |  |  |  |  |
| 22 | REN42F10_F | AATGTCTCATCGGAGGAA | 7.4 | 52 | 4 | 210-216 | 58.2 | 51.7 |
|  | REN42F10_R | AGACCGAGCTTTCATCAGA |  |  |  |  |  |  |
| 22 | FH3411_F | CAAATGGAAGTGAAAAGAAAGC | 39.6 | 60 | 17 | 268-332 | 90.2 | 88.5 |
|  | FH3411_R | ATATGTTCTGGCTGGATCATTC |  |  |  |  |  |  |
| 37 | FH3272_F | GGATCCGGGATAGAGCAG | 5.0 | 60 | 15 | 295-399 | 70.4 | 69.6 |
|  | FH3272_R | CTTCCCCAATCTTCCTTGTC |  |  |  |  |  |  |
| 37 | FH2532_F | CACGCAGAAAGGCAGAAAG | 25.2 | 58 | 14 | 332-432 | 70.5 | 67.3 |
|  | FH2532_R | TTTCCATAGTGGCTGCATCA |  |  |  |  |  |  |
