# Supplementary material for: A One Base Pair Deletion in the Canine ATP13A2 Gene Causes Exon Skipping and Late-Onset Neuronal Ceroid Lipofuscinosis in the Tibetan Terrier
Source: PLoS Genet. 2011 Oct 13;7(10):e1002304. doi: 10.1371/journal.pgen.1002304 (PMC3192819; doi:10.1371/journal.pgen.1002304)
Supplement: Table S2 — Tibetan terrier families used for linkage analysis. For linkage analysis 20 NCL-affected and 87 NCL-unaffected Tibetan terriers were genotyped in three families enclosing a total of 107 Tibetan terriers. (DOC) [file pgen.1002304.s007.doc]

| Family | NCL-affected | NCL-affected (genotyped) | NCL-unaffected | NCL-unaffected (genotyped) | Total | Total (genotyped) |
| --- | --- | --- | --- | --- | --- | --- |
| 1 | 5 | 5 | 82 | 60 | 87 | 65 |
| 2 | 13 | 11 | 43 | 13 | 56 | 24 |
| 3 | 4 | 4 | 22 | 14 | 26 | 18 |
| total | 22 | 20 | 146 | 87 | 171 | 107 |
