# Supplementary material for: A One Base Pair Deletion in the Canine ATP13A2 Gene Causes Exon Skipping and Late-Onset Neuronal Ceroid Lipofuscinosis in the Tibetan Terrier
Source: PLoS Genet. 2011 Oct 13;7(10):e1002304. doi: 10.1371/journal.pgen.1002304 (PMC3192819; doi:10.1371/journal.pgen.1002304)
Supplement: Table S3 — Chromosome-wide error probabilities (pZmean, pLOD) for multipoint non-parametric linkage analysis in 107 Tibetan terriers for the NCL-phenotype using 28 microsatellites. (DOC) [file pgen.1002304.s008.doc]

| Marker | Zmean | P-value | LOD | P-value | CFA |
| --- | --- | --- | --- | --- | --- |
| AHT111 | 1.16 | 0.12 | 0.41 | 0.08 | 2 |
| 2_81.88 | 1.75 | 0.04 | 0.64 | 0.04 | 2 |
| FH2026 | 1.75 | 0.04 | 0.64 | 0.04 | 2 |
| 2_82.45 | 1.85 | 0.03 | 0.70 | 0.04 | 2 |
| 2_82.86 | 1.92 | 0.03 | 0.73 | 0.03 | 2 |
| 2_83.22 | 3.20 | 0.0007 | 1.14 | 0.011 | 2 |
| 2_83.77 | 3.52 | 0.0002 | 1.2 | 0.009 | 2 |
| 2_84.19 | 3.76 | 0.00008 | 1.24 | 0.008 | 2 |
| 2_84.23 | 3.78 | 0.00008 | 1.25 | 0.008 | 2 |
| 2_85.05 | 4.09 | 0.00002 | 1.26 | 0.008 | 2 |
| 2_85.95 | 4.60 | <0.00001 | 1.33 | 0.007 | 2 |
| 2_86.44 | 4.55 | <0.00001 | 1.32 | 0.007 | 2 |
| 2_87.24 | 4.45 | <0.00001 | 1.32 | 0.007 | 2 |
| 2_88.00 | 4.28 | <0.00001 | 1.29 | 0.007 | 2 |
| FH3218 | 0.03 | 0.5 | 0.01 | 0.4 | 8 |
| C08.410 | -0.05 | 0.5 | -0.01 | 0.6 | 8 |
| C08.618 | 0.24 | 0.4 | 0.11 | 0.2 | 8 |
| REN248C19 | -0.00 | 0.5 | -0.00 | 0.5 | 18 |
| REN47J11 | -0.01 | 0.5 | -0.00 | 0.5 | 18 |
| REN50L03 | -0.07 | 0.5 | -0.00 | 0.5 | 18 |
| FH2429 | -0.34 | 0.6 | -0.05 | 0.7 | 18 |
| REN49F22 | -0.84 | 0.8 | -0.13 | 0.8 | 22 |
| REN68D20 | -0.85 | 0.8 | -0.13 | 0.8 | 22 |
| REN42F10 | -0.86 | 0.8 | -0.13 | 0.8 | 22 |
| FH3411 | -0.86 | 0.8 | -0.12 | 0.8 | 22 |
| FH3272 | -0.23 | 0.6 | -0.02 | 0.6 | 37 |
| FH2532 | -0.43 | 0.7 | -0.06 | 0.7 | 37 |
| REN157D13 | -0.46 | 0.7 | -0.07 | 0.7 | 37 |
