# Supplementary material for: A One Base Pair Deletion in the Canine ATP13A2 Gene Causes Exon Skipping and Late-Onset Neuronal Ceroid Lipofuscinosis in the Tibetan Terrier
Source: PLoS Genet. 2011 Oct 13;7(10):e1002304. doi: 10.1371/journal.pgen.1002304 (PMC3192819; doi:10.1371/journal.pgen.1002304)
Supplement: Table S4 — Summary results for the SNPs used for association analysis. SNP accession numbers, positions and alleles genotyped for 376 Tibetan terriers with their minor allele frequencies (MAF) and P-values for genotypic association with NCL. (DOC) [file pgen.1002304.s009.doc]

| CFA | SNP accession numbers | Position in bp | Allele 1 | Allele 2 | MAF | P-value |
| --- | --- | --- | --- | --- | --- | --- |
| 2 | BICF2P1245823 | 82188014 | C | T | 0.50 | 0.0000002 |
| 2 | BICF2S22913250 | 82585043 | C | T | 0.44 | 0.0000002 |
| 2 | TIGRP2P32267_rs8882829 | 82706430 | C | T | 0.11 | 0.006 |
| 2 | BICF2P506477 | 83190821 | C | G | 0.18 | 0.07 |
| 2 | BICF2S23719003 | 83944434 | C | A | 0.34 | 0.0000002 |
| 2 | BICF2P340010 | 84091683 | A | G | 0.43 | 0.0000002 |
| 2 | BICF2P573921 | 86179624 | C | T | 0.16 | 0.0008 |
| 8 | BICF2P1128894 | 4184588 | T | A | 0.37 | 0.000001 |
| 8 | BICF2P221695 | 61691605 | G | T | 0.13 | 1 |
| 12 | BICF2G630121377 | 60205389 | C | T | 0.48 | 0.00003 |
| 12 | BICF2P1349378 | 61277916 | A | G | 0.15 | 0.1 |
| 12 | BICF2G630120781 | 61316848 | A | T | 0.21 | 0.09 |
| 18 | BICF2P386921 | 48628596 | A | G | 0.39 | 0.00005 |
