# Supplementary material for: A One Base Pair Deletion in the Canine ATP13A2 Gene Causes Exon Skipping and Late-Onset Neuronal Ceroid Lipofuscinosis in the Tibetan Terrier
Source: PLoS Genet. 2011 Oct 13;7(10):e1002304. doi: 10.1371/journal.pgen.1002304 (PMC3192819; doi:10.1371/journal.pgen.1002304)
Supplement: Table S5 — Candidate genes evaluated for late-onset NCL in Tibetan terriers. Gene name, annotation by NCBI or Ensembl, position in mega bases (Mb) and involvement in disease/function of genes in human. (DOC) [file pgen.1002304.s010.doc]

| Gene name | Full gene name | *Canis lupus familiaris* genome build 2.1 | Position of gene in Mb | Function |
| --- | --- | --- | --- | --- |
| *PINK1* | *PTEN induced putative kinase 1* | ENSCAFG00000015045  (Ensembl) | 81,170,900- 81,187,983 | Parkinson |
| *H2A* | *H2A histone family, member Z* | LOC478215 | 83,849,824- 83,850,706 | Systemic lupus erythematosus |
| *PADI3* | *peptidyl arginine deiminase, type III* | LOC478216 | 83,863,546-83,890,271 | Keratinocyten |
| *PADI2* | *peptidyl arginine deiminase, type II* | LOC487414 | 83,964,490-84,041,410 | Neurodegenerative human disorders, Alzheimer |
| *SDHB* | *succinate dehydrogenase complex, subunit B, iron sulfur (Ip)* | LOC478217 | 84,054,661-84,086,382 | Alzheimer, Parkinson |
| *ATP13A2* | *ATPase type 13A2* | ENSCAFG00000015840 (Ensembl) | 84,009,000-84,111,000 | Parkinson |
| *NECAP2* | *NECAP endocytosis associated 2* | LOC478218 | 84,172,725-84,185,772 | Endocytosis |
| *MAPK*  *PM20/PM21* | *putative MAPK activating protein PM20,PM21 isoform 2* | LOC608518 | 84,223,233-84,247,082 | Cellular signal transduction |
| *FBXO42* | *F-box protein 42* | LOC478219 | 84,254,604-84,354,033 |  |
| *REM2* | *GTP-binding protein REM2* | LOC487419 | 84,364,611-84,369,548 |  |
| *SLC25A34* | *solute carrier family 25, member 34* | LOC608629 | 84,758,707-84,762,000 | Mitochondrial carrier protein in central nervous system |
| *CASP9* | *caspase 9, apoptosis-related cysteine peptidase* | *CASP9* | 84,932,236-84,952,107 | Neuronal apoptosis, Alzheimer, Parkinson |
| *TMEM51* | *transmembrane protein 51* | LOC607267 | 85,149,882-85,153,510 |  |
| *CLCN6* | *chloride channel 6* | LOC478229 | 87,295,994-87,328,946 | Late-onset NCL in human |
