# Supplementary material for: A One Base Pair Deletion in the Canine ATP13A2 Gene Causes Exon Skipping and Late-Onset Neuronal Ceroid Lipofuscinosis in the Tibetan Terrier
Source: PLoS Genet. 2011 Oct 13;7(10):e1002304. doi: 10.1371/journal.pgen.1002304 (PMC3192819; doi:10.1371/journal.pgen.1002304)
Supplement: Table S6 — Primer pairs for cDNA amplification and the genomic regions of the 14 canine candidate genes. The primer pairs are given with their sequences, annealing temperature (AT), product size in base pairs (bp) and the target position. (DOC) [file pgen.1002304.s011.doc]

| Name | Primer sequence (5’-3’) | AT | Product size (bp) | Target |
| --- | --- | --- | --- | --- |
| PINK1_F1 | ACCGCTACCGCTTCTTCC | 60 | 538 | exon 1 |
| PINK1_R1 | CGACTAGCTGGGACGAGTTC |  |  | exon 3 |
| PINK1_F2 | GAACTCGTCCCAGCTAGTCG | 60 | 698 | exon 3 |
| PINK1_R2 | CAGCTGAGCCTCCTGGTAAC |  |  | exon 7 |
| PINK1_F3 | GTGGGTACGCTTGCCTATGA | 60 | 663 | exon 7 |
| PINK1_R3 | CGCAGTTAGACGATGGACCT |  |  | exon 9-3’UTR |
| PINK1_Ex1F | CTGAAACGACGTTCTGAGCA | 60 | 690 | 5’UTR |
| PINK1_Ex1R | CACCTGGATCTCCCGACA |  |  | intron 1 |
| H2A_F1 | TTCCGTCCTTTCCTTATTGG | 59 | 385 | exon 1-3’UTR |
| H2A_R1 | TTTAATTAGCTTTCCAACCAACC |  |  | exon 1 |
| H2A_F2 | GCAGAAATTTGGTTGGTTGG | 60 | 540 | exon 1 |
| H2A_R2 | GGTGCGATGACGGAGTAGG |  |  | exon 1-5’UTR |
| PADI3_F1 | CGTGCTATCTGGAACCTTGG | 60 | 597 | 5’UTR |
| PADI3_R1 | TCCAGGAAAAGCAGAAGTGG |  |  | exon 4 |
| PADI3_F2 | CCTGGAAGAGCTTGAAGCAG | 60 | 658 | exon 4 |
| PADI3_R2 | CCGATCTTCACCGACACC |  |  | exon 9 |
| PADI3_F3 | GTGCTGGGCGTCATAATCC | 61 | 578 | exon 9 |
| PADI3_R3 | TACGCGGTGCTCTACCTCAC |  |  | exon 14 |
| PADI3_F4 | TCGCAGTTCAGGTCACAATC | 60 | 450 | exon 13 |
| PADI3_R4 | CTTTTAAACCGCACGACCTG |  |  | exon 16 |
| PADI2_F1 | TGAGGCCCAGACCTTCAG | 59 | 763 | exon 1 |
| PADI2_R1 | TTCTCATCGTTCAGGTCCTC |  |  | exon 4 |
| PADI2_F2 | CTGCTGGTGAACTGTGACC | 58 | 698 | exon 4 |
| PADI2_R2 | TTCACTGGGAAGTCCTTCAG |  |  | exon 9 |
| PADI2_F3 | AAGGCTTCCCTGTGGTACTG | 58 | 398 | exon 9 |
| PADI2_R3 | CGAGGTACTGGCCATGAG |  |  | exon 11 |
| PADI2_F4 | CTCTACTCGGACTGGCTGAC | 58 | 672 | exon 10 |
| PADI2_R4 | AGCCACAAAATCAGCTATGG |  |  | exon 14 |
| SDHB_F1 | GTGACGTCAGGAGCCAAG | 58 | 741 | exon 1 |
| SDHB_R1 | CTTCTTCAAATAAGGTTCAATGG |  |  | exon 5 |
| SDHB_F2 | ACACCAACCTCAGCAAAGTC | 58 | 495 | exon 4 |
| SDHB_R2 | CCTGCACATTTCTCTTCAGC |  |  | exon 8-3’UTR |
| NECAP2_F1 | TCGAGATGGAGGAGGGCG | 60 | 689 | exon 1 |
| NECAP2_R1 | GTCTGGGTTCTGGGCTTG |  |  | exon 5 |
| NECAP2_F2 | GGGCATTTATTGGAATTGG | 58 | 837 | exon 4 |
| NECAP2_R2 | CACACTCAACCCCTTCTTTG |  |  | exon 10-3’UTR |
| MAPK_F1 | GGCGAGTAAGATGGAAGATG | 58 | 551 | exon 1 |
| MAPK_R1 | GGGGTCACAGTGCAAGTAAG |  |  | exon 3 -3’UTR |
| MAPK_F2 | ACAGCTGGACTTGAGCAGAG | 58 | 693 | exon 3 -3’UTR |
| MAPK_R2 | GAAGATGCCCTGGGACTTAG |  |  | exon 3 -3’UTR |
| MAPK_F3 | CCCAGGACAGATGAGCAC | 58 | 741 | exon 3 -3’UTR |
| MAPK_R3 | AGTGCAATGGAAGAGACCAG |  |  | exon 3 -3’UTR |
| FBXO42_F1 | GTTTGAGCGGAGGAAGATGG | 788 | 60/61 | exon 1 |
| FBXO42_R1 | GATATGGGCTTGGTCGTGTC |  |  | exon 5 |
| FBXO42_F2 | TCGTGTACAAGGACCTGCTG | 59 | 372 | exon 5 |
| FBXO42_R2 | CCACATCCTCCAAGGATTAAG |  |  | exon 8 |
| FBXO42_F3 | TCCTTGACCTTGAGCAGTGG | 60/61 | 554 | exon 7 |
| FBXO42_R3 | AGACAAATTGCCACCATTGAG |  |  | exon 10 |
| FBXO42_F4 | AAGGCAGACCCCCTCAAG | 60 | 496 | exon 10 |
| FBXO42_R4 | CAGAGGAGGGCTTAGTGCTG |  |  | exon 10 |
| FBXO42_F5 | CCCTTGCAGGAGCTGTCTC | 60/61 | 586 | exon 10 |
| FBXO42_R5 | TTCTCAGTCCAAATGCTTGC |  |  | exon 10-3’UTR |
| REM2_F1 | GTCGGAGAGCAGGTTGAGAG | 60 | 778 | exon 1 |
| REM2_R1 | CGCCTGAGAGTTGGGAATAG |  |  | exon 5-3’UTR |
| SLC25A35_F1 | AGCAGAGCTGGGGGACTC | 60 | 747 | exon 1 |
| SLC25A35_R1 | AGCTGAGCCGACCATGAC |  |  | exon 3 |
| SLC25A35_F2 | GTGCCTTGGAGACCATCTG | 60 | 733 | exon 3 |
| SLC25A35_R2 | TGCCCTGGAGCAGTTATTATG |  |  | exon 5 |
| TMEM51_F1 | ACGTGGCACTAGGGATTTTG | 60 | 689 | 5’UTR |
| TMEM51_R1 | CGTTTGGCCAACTTGGAG |  |  | exon 2 |
| TMEM51_F2 | TTAAATCCGAAAAGCTGCAC | 58 | 549 | exon 2 |
| TMEM51_R2 | CAAAAGGCCTGTCTTGTTCTC |  |  | exon 2 |
| TMEM51_F3 | TCAGGACAGGACACCTAGCAG | 60 | 498 | exon 2 |
| TMEM51_R3 | TGATCTGTTTTGTGCAGATTAGC |  |  | exon 2-3’UTR |
| CASP9_F1 | GAGCCGCGAGCTCTTCAC | 61 | 373 | exon 1 |
| CASP9_R1 | CGATGGTCTGACAACCTGTG |  |  | exon 2 |
| CASP9_F2 | GCTGACTCCAGGAAAACTCG | 60 | 550 | exon 2 |
| CASP9_R2 | AAAGAAGAGCTTGGGCTTCC |  |  | exon 6 |
| CASP9_F3 | GGCACAGATGGATGTCCTG | 60 | 356 | exon 6 |
| CASP9_R3 | ACACCATCCAAGGTCTCCAC |  |  | exon 8 |
| CASP9_F4 | GACGCCGTGTCTAGTTTGC | 60 | 711 | exon 7 |
| CASP9_R4 | TCACTGCTCAGACCAAGGTG |  |  | exon 9-3’UTR |
| CLCN6_ F1 | GGAGGAGGATGATGAGATCC | 58 | 845 | exon 2 |
| CLCN6_ R1 | TAAACTCGCCAAAGTTCAGC |  |  | exon 11 |
| CLCN6_ F2 | TTCACCCTCAACTTCTTTCG | 58 | 773 | exon 11 |
| CLCN6_ R2 | GATCAGGATGACCGTGAGAC |  |  | exon 16 |
| CLCN6_ F3 | ACATCTATTCGGGGACCTTC | 58 | 722 | exon 16 |
| CLCN6_ R3 | GACGAGCAGGGTGACAAG |  |  | exon 20 |
| CLCN6_ F4 | ACCCCAACCTATACCCTGAC | 58 | 723 | exon 20 |
| CLCN6_R4 | AAGCAGGGAGCAGGAAAC |  |  | exon 23-3’UTR |
