# Supplementary material for: A One Base Pair Deletion in the Canine ATP13A2 Gene Causes Exon Skipping and Late-Onset Neuronal Ceroid Lipofuscinosis in the Tibetan Terrier
Source: PLoS Genet. 2011 Oct 13;7(10):e1002304. doi: 10.1371/journal.pgen.1002304 (PMC3192819; doi:10.1371/journal.pgen.1002304)
Supplement: Table S7 — Single nucleotide polymorphisms detected in coding and untranslated sequences of the evaluated genes for NCL in Tibetan terriers. SNP nomenclature, position and possible amino acid exchange are given. (DOC) [file pgen.1002304.s012.doc]

| Gene name | SNP name | SNP position | Amino acid exchange |
| --- | --- | --- | --- |
| *PINK1* | DN424117:c.406C>T | Exon 4 | - |
| *H2A* | XM_859525:c.347G>T | 3’UTR | - |
| *PADI3* | XM_535391:c.1786C>T | Exon 16 | tyrosine>histidine |
|  | XM_535391:c.2032G>T | 3’UTR | - |
| *PADI2* | XM_544539:c.840T>C | Exon 8 | - |
|  | XM_544539:c.1587C>T | Exon 12 | - |
| *SDHB* | - | - | - |
| *ATP13A2* | cDNA:c.1620delG | Exon 16 | - |
| *NECAP2* | XM_535393:c.176G>A | Exon 2 | - |
| *MAPK*  *PM20/PM21* | XM_846908:c.766T>C | 3’UTR | - |
| *FBXO42* | - | - | - |
| *REM2* | rs22840069 | Exon 4 | - |
| *SLC25A34* | XM_847049:c.109G>A | 5’UTR | - |
|  | XM_847049:c.717T>C | Exon 3 | - |
|  | XM_847049:c1030A>G | Exon 5 | threonine>alanine |
|  | XM_847049:c.1037T>C | Exon 5 | - |
|  | XM_847049:c.1101A>G | Exon 5 | - |
| *CASP9* | NM_001031633:c213C>T | Exon 2 | serine>proline |
| *TMEM51* | XM_843883:c.702A>G | Exon 2 | - |
|  | XM_843883:c.705A>G | Exon 2 | - |
| *CLCN6* | XM_535404:c.2136T>C | Exon 19 | - |
|  | XM_535404:c.2706A>C | 3’UTR | - |
|  | XM_535404:c.2761A>G | 3’UTR | - |
