# Supplementary material for: A One Base Pair Deletion in the Canine ATP13A2 Gene Causes Exon Skipping and Late-Onset Neuronal Ceroid Lipofuscinosis in the Tibetan Terrier
Source: PLoS Genet. 2011 Oct 13;7(10):e1002304. doi: 10.1371/journal.pgen.1002304 (PMC3192819; doi:10.1371/journal.pgen.1002304)
Supplement: Table S8 — Genotyping 376 Tibetan terriers for the canine MAPK PM20/PM21 c.766T>C SNP. (DOC) [file pgen.1002304.s013.doc]

| *MAPK PM20/PM21* XM_846908:c.766T>C | NCL-affected (n=24) | NCL-carrier (n=30) | Unaffected or unknown (n=322) |
| --- | --- | --- | --- |
| C/C | 22 | 3 | 9 |
| C/T | 2 | 27 | 103 |
| T/T | - | - | 210 |
