# Supplementary material for: A One Base Pair Deletion in the Canine ATP13A2 Gene Causes Exon Skipping and Late-Onset Neuronal Ceroid Lipofuscinosis in the Tibetan Terrier
Source: PLoS Genet. 2011 Oct 13;7(10):e1002304. doi: 10.1371/journal.pgen.1002304 (PMC3192819; doi:10.1371/journal.pgen.1002304)
Supplement: Table S9 — Primer pairs for cDNA amplification of the canine ATP13A2 gene and its genomic DNA surrounding exon 16. The primer pairs are given with their sequences, annealing temperature (AT), product size in base pairs (bp) and the target position. (DOC) [file pgen.1002304.s014.doc]

| Name | Primer sequence (5’-3’) | AT | Product size (bp) | Target |
| --- | --- | --- | --- | --- |
| ATP13A2_F5 | CGGCTATGGGACCCTGAC | 59 | 811 | exon 2 |
| ATP13A2_R5 | CTGCACCATGTCCCTCAG |  |  | exon 10 |
| ATP13A2_F1 | GAGGATAATTCCTGGCAGCTC | 60 | 834 | exon 4 |
| ATP13A2_R1 | GAGGGTCCCACAGAAGAGTG |  |  | exon 12 |
| ATP13A2_F6 | ACTATGGGTTCCAGGCCTTC | 60 | 824 | exon 9 |
| ATP13A2_R6 | CTTGTCAAAACACACGAGCTG |  |  | exon 15 |
| ATP13A2_F7 | TGCCTCTGAATGAGATCGTG | 60 | 687 | exon 15 |
| ATP13A2_R7 | TGTAGCTCTGCAGCATCTGG |  |  | exon 19 |
| ATP13A2_F8 | AGATGCTGCAGAGCTACACG | 60 | 820 | exon 19 |
| ATP13A2_R8 | ACGGAGATGAACTGGGTCAG |  |  | exon 25 |
| ATP13A2_F9 | GTACTGCGTGGGCATGTG | 60 | 566 | exon 24 |
| ATP13A2_R9 | GGTACTGGAAGCCAGACAGG |  |  | exon 27 |
| ATP13A2_F3 | CCCATGGTCATCAGGTGAG | 60 | 530 | exon 24 |
| ATP13A2_R3 | AGCAGCTTGAAGCAGGTGTC |  |  | exon 28 |
| ATP13A2_F10 | CTGCCCAACTATGAGAACACC | 60 | 700 | exon 27 |
| ATP13A2_R10 | CGTGGTGCCACATAGGAGAC |  |  | exon 29 (3’UTR) |
| ATP13A2_F4 | CTCCTGGGCTCCATCCTG | 60 | 474 | exon 28 |
| ATP13A2_R4 | TCGTCTACACAGGATGTCTGAAG |  |  | exon 29 (3’UTR) |
| ATP13A2_mutF | GACCTGCCGTAGGGTGAAG | 60 | 635 | intron 15 |
| ATP13A2_mutR | AAGCTTCCTTCCTGGGCTAC |  |  | intron 16 |
