# Supplementary material for: A One Base Pair Deletion in the Canine ATP13A2 Gene Causes Exon Skipping and Late-Onset Neuronal Ceroid Lipofuscinosis in the Tibetan Terrier
Source: PLoS Genet. 2011 Oct 13;7(10):e1002304. doi: 10.1371/journal.pgen.1002304 (PMC3192819; doi:10.1371/journal.pgen.1002304)
Supplement: Table S10 — Dog breeds other than Tibetan terrier genotyped for the ATP13A2 c.1620delG mutation. (DOC) [file pgen.1002304.s015.doc]

| Breeds | Number of dogs (n=144) |  | *ATP13A2* c.1620delG | | |
| --- | --- | --- | --- | --- | --- |
|  |  |  | G/G | G/delG | delG/delG |
| German wirehaired pointer | 16 |  | 16 | - | - |
| Irish wolfhound | 16 |  | 16 | - | - |
| German shepherd dog | 16 |  | 16 | - | - |
| Dalmatian | 16 |  | 16 | - | - |
| Newfoundland dog | 8 |  | 8 | - | - |
| Airedale terrier | 8 |  | 8 | - | - |
| Bernese mountain dog | 16 |  | 16 | - | - |
| Large Swiss mountain dog | 16 |  | 16 | - | - |
| Entlebuch mountain dog | 4 |  | 4 | - | - |
| Appenzell mountain dog | 4 |  | 4 | - | - |
| German Pinscher | 14 |  | 14 | - | - |
