# Supplementary material for: A One Base Pair Deletion in the Canine ATP13A2 Gene Causes Exon Skipping and Late-Onset Neuronal Ceroid Lipofuscinosis in the Tibetan Terrier
Source: PLoS Genet. 2011 Oct 13;7(10):e1002304. doi: 10.1371/journal.pgen.1002304 (PMC3192819; doi:10.1371/journal.pgen.1002304)
Supplement: Supplementary file 13 [file pgen.1002304.s003.doc]

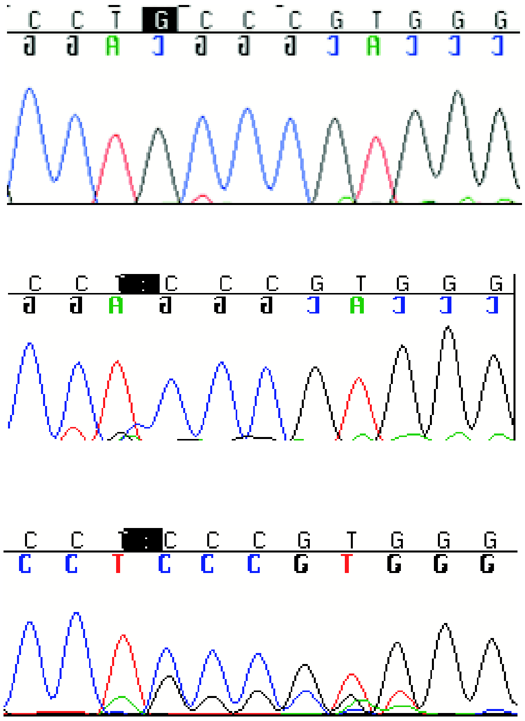

Non-affected wild type

NCL-affected

NCL-carrier

| | | | | |
|---|---|---|---|---|
| Reference sequence NW_876293 | AGAGCCCCGCCGCCT | G | CCCGTGGGGCCCCTG | |
| Tibetan terrier NCL-unaffected | AGAGCCCCGCCGCCT | G | CCCGTGGGGCCCCTG | |
| Tibetan terrier NCL-affected | AGAGCCCCGCCGCCT | : | CCCGTGGGGCCCCTG | |
| | -15 bp | 96 | | +15 bp |
